# Optical Coherence Tomography Angiography

**DOI:** 10.1155/2018/7140164

**Published:** 2018-06-06

**Authors:** Stephen G. Schwartz, Harry W. Flynn, Andrzej Grzybowski, Avinash Pathengay, Ingrid U. Scott

**Affiliations:** ^1^Department of Ophthalmology, Bascom Palmer Eye Institute, University of Miami Miller School of Medicine, Miami, FL, USA; ^2^Institute for Research in Ophthalmology, Poznan, Poland; ^3^Department of Ophthalmology, University of Warmia and Mazury, Olsztyn, Poland; ^4^Retina and Uveitis Department, LV Prasad Eye Institute, GMR Varalakshmi Campus, Visakhapatnam, Andhra Pradesh, India; ^5^Department of Ophthalmology, Penn State College of Medicine, Hershey, PA, USA; ^6^Department of Public Health Sciences, Penn State College of Medicine, Hershey, PA, USA

Optical coherence tomography angiography (OCT-A) is a relatively new, noninvasive outpatient imaging test that provides both structural and functional information about the macula and midperipheral retina. OCT-A is complementary to traditional imaging modalities, such as fundus photography, fluorescein angiography (FA), and spectral domain optical coherence tomography (SD-OCT).

Advantages of OCT-A include visualization of vascular flow signal,* en face* imaging, no need for dye injection, and segmentation of the posterior segment structures from the vitreomacular interface, through the retinal layers, and to the choroid. Disadvantages of OCT-A include increased costs and longer acquisition times than SD-OCT. Because of the longer acquisition times, it may be difficult to obtain high-quality OCT-A images in eyes with poor visual acuity that cannot fixate well.

The use of OCT-A is currently best established in the care of patients with retinal vascular diseases, choroidal vascular diseases, and ophthalmic oncology. Its role appears to be emerging in patients with other macular and retinal diseases (including uveitis) as well as optic nerve diseases and glaucoma.

This special issue, which had opened for 6 months in the second half of 2017, focuses on various clinical applications of OCT-A.

V. M. Villegas et al. present two patients with choroidal nevi, including one halo nevus, and report decreased vascular flow signal in most or all layers on OCT-A. This decreased vascular flow signal is proposed to be due to blockage from the choroidal nevus, true diminished blood flow (ischemia), or unknown causes. Because choroidal melanoma frequently demonstrates increased vascularity on FA, the authors propose that OCT-A might represent a noninvasive test to screen suspicious nevi for evidence of early malignant transformation.

V. Shah et al. report one patient with a unilateral congenital retinal macrovessel in the macula. OCT-A demonstrates replacement of the normal foveal avascular zone (FAZ) by abnormal vascular bifurcations, yet this disturbance of the FAZ is associated with relatively normal foveal anatomy, as imaged by swept-source OCT, and a best-corrected visual acuity of 20/20.

B. M. Hampton et al. present one patient with bilateral choroidal neovascularization (CNV) due to punctate inner choroidopathy (PIC). In this patient, OCT-A demonstrates bilateral submacular abnormal vessels consistent with CNV, and FA demonstrates late vascular leakage, confirming the diagnosis.

V. M. Villegas and J. L. Kovach report one patient with bilateral macular telangiectasia type 2 (MacTel2) and unilateral subretinal neovascularization (SNV). In the eye with SNV, OCT-A demonstrates abnormal submacular vessels consistent with SNV. In the fellow eye, OCT-A demonstrates abnormal vessels temporal to the center of the macula consistent with nonproliferative MacTel2.

P. Shah et al. present two patients with acute central retinal artery occlusion (CRAO), including one with cilioretinal artery sparing, imaged with both FA and OCT-A. In both patients, the images obtained by FA and OCT-A are very similar. Because of this similarity, the authors propose that patients with acute CRAO in whom OCT-A can be obtained might not require additional imaging with FA.

M. Kaya et al. report one patient with chronic combined cilioretinal artery occlusion and central retinal vein occlusion (CRVO). In this patient, FA performed 10 months after the combined occlusion is relatively normal but OCT-A demonstrates a wedge-shaped area of decreased vascular flow signal consistent with the cilioretinal artery occlusion. The authors propose that different imaging studies may be relatively more useful in the acute and chronic phases of this disease.

S. Wu et al. present a somewhat similar patient with combined CRAO and CRVO. In this patient, symptoms began immediately following cataract surgery with retrobulbar anesthesia. OCT-A demonstrates profound decreased vascular flow signal in the superficial and deep retinal plexuses but relatively normal vascular flow signal in the choriocapillaris and choroid.

T. Y. A. Liu et al. report one patient with CNV due to presumed ocular histoplasmosis syndrome (POHS). Three monthly injections of intravitreal bevacizumab were given. On follow-up examinations up to 6 months, CNV activity is not detectable by FA or SD-OCT but is detectable by OCT-A. The authors propose that OCT-A might be more sensitive than FA for this indication.

H. Hamoudi et al. present one patient with unilateral Purtscher retinopathy following thoracic trauma sustained in a motor vehicle accident. OCT-A demonstrates decreased vascular flow signal in the superficial and deep retinal capillary plexuses of the affected eye and is normal in the fellow eye.

V. S. Chang et al. report one patient with unilateral retinal arterial macroaneurysm (RAM) treated with two injections of intravitreal bevacizumab. At presentation, OCT-A demonstrated increased vascular flow signal in the walls of the RAM. After treatment with bevacizumab was initiated, follow-up OCT-A studies demonstrate progressive reduction in the vascular flow signal within the walls of the RAM, suggesting progressive sclerosis of the lesion.

A. Fukutomi et al. present one patient with nonischemic CRVO that progressed to ischemic CRVO. At presentation, OCT-A demonstrated some reduction in vascular flow signal in the small capillaries but relatively preserved vascular flow signal surrounding the FAZ. Subsequent OCT-A studies demonstrate progressive expansion of the area of decreased vascular signal flow, consistent with progressive expansion of capillary nonperfusion.

In summary, this special issue contains many interesting case reports, which collectively illustrate many uses of OCT-A in patients with retinal vascular disease, choroidal vascular disease, and ocular oncology.

